# Double Seronegative Neuromyelitis Optica Spectrum Disorder with Longitudinally Extensive Transverse Myelitis and Optic Neuritis: A Challenging Case Report

**DOI:** 10.7759/cureus.57044

**Published:** 2024-03-27

**Authors:** Goh Chon Han, Tajunisah Iqbal, Gowri Supramaniam

**Affiliations:** 1 University of Malaya Eye Research Centre, Department of Ophthalmology, Faculty of Medicine, Universiti Malaya, Kuala Lumpur, MYS; 2 Department of Ophthalmology, Hospital Tuanku Jaafar Seremban, Negeri Sembilan, MYS

**Keywords:** double seronegative optic neuritis, double seronegative nmosd, multiple sclerosis and other demyelinating disorders, neuromyelitis optica spectrum disorder (nmosd), neuromyelitis optica spectrum disorder, plasma exchange therapy, therapeutic plasma exchange (tpe), anti-aquaporin 4 antibody, igg autoantibodies to aquaporin 4, bilateral optic neuritis

## Abstract

Neuromyelitis optica spectrum disorder (NMOSD) is a rare antibody-mediated neuroinflammatory disease of the central nervous system, typically manifesting in the optic nerves, spinal cord, and other regions of the central nervous system. We hereby report a case of a 16-year-old girl who presented with a six-month history of transverse myelitis with an acute episode of bilateral retrobulbar optic neuritis. MRI revealed patchy contrast enhancements over bilateral retrobulbar intraorbital optic nerves together with long-segment spinal cord hyperintensities (C2 to T2 level). Visual evoked potential testing during the acute presentation showed the absence of P100 bilaterally. However, both serum AQP4-IgG and MOG-IgG were reported to be negative. Despite remarkable improvement in bilateral optic nerve functions, she continued to have disabling bilateral lower limb spasticity, contractures, and loss of bilateral lower limb sensation after five cycles of plasma exchange. This case summarizes the challenges to diagnosing double seronegative NMOSD and its immediate therapeutic significance.

## Introduction

Neuromyelitis optica spectrum disorder (NMOSD) is a rare antibody-mediated neuroinflammatory disease of the central nervous system, typically manifesting in the optic nerves, the spinal cord, and other central nervous system regions [1-3]. The prevalence of NMOSD falls within a range of 0.5-4 per 100,000 individuals [3]. Classically, NMOSD is associated with aquaporin-4 antibody (AQP4-IgG) or myelin oligodendrocyte glycoprotein antibody (MOG-IgG) [4-6]. However, there is a small subset of patients categorized as having double seronegative-NMOSD (DN-NMOSD) who are seronegative for these two antibodies despite using the most sensitive laboratory techniques [6]. The underlying disease mechanism of DN-NMOSD is heterogenous and has no diagnostic marker [6]. This case report illustrates a rare case of DN-NMOSD in a 16-year-old girl, highlighting its diagnostic challenges and therapeutic significance. 

## Case presentation

We report a 16-year-old girl who was referred for a sudden bilateral blurring of vision persisting for five days. She also experienced muscle weakness and loss of sensation in bilateral lower limbs over the past five months. She experienced easy fatigability and shivering of bilateral lower limbs while climbing stairs during the first two weeks after the onset of symptoms. It worsened into frequent falls and requiring assistance during walking in a month. There were no related trauma or constitutional symptoms.

Neurological examination revealed bilateral spastic tetraparesis with reduced crude touch, pain, temperature, and proprioception at the level of C2-T2 and T5-L4. Clonus was noted over bilateral lower limbs. Hyperreflexia was noted in all limbs. Barbinski's reflexes were upgoing with a fanning pattern in bilateral lower limbs.

**Figure 1:**
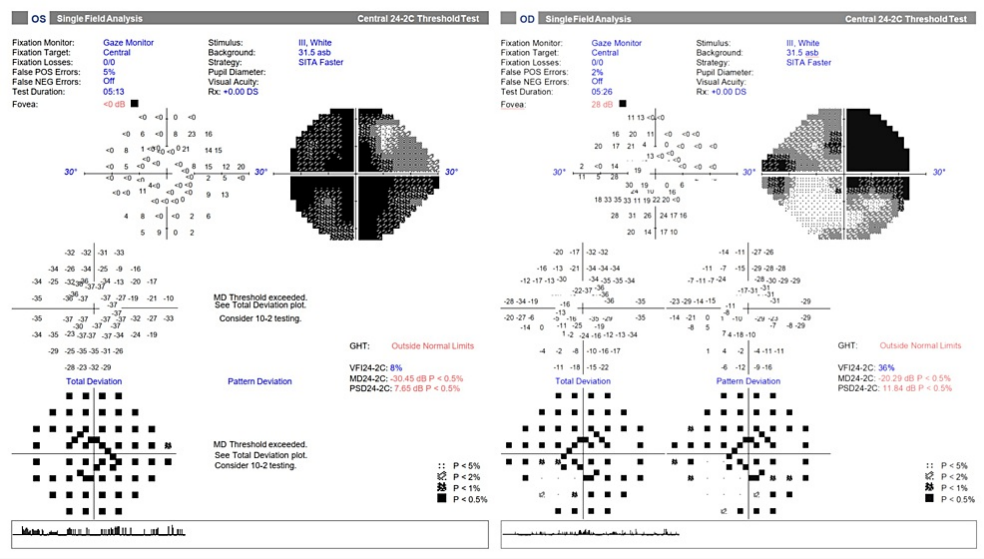
24-2 Humphrey visual field testing of the left eye (OS) and right eye (OD) during the initial presentation

Her best-corrected visual acuity during the initial presentation was 6/36 for the right eye and 6/60 for the left eye (Table 1). Her bilateral myopia of -5.00D was fully correctable with refractive spectacles before the onset of visual symptoms. Visual field testing revealed diffuse visual field loss over the left eye (OS), and superiotemporal quadrant field loss with nasal step visual field defects over the right eye (OD) (Figure 1). There was no evidence of acute or chronic optic nerve head swelling. Other cranial nerve examinations were normal.

**Table 1:** Summary of the optic nerve function findings

Parameter	Right Eye	Left Eye
Best Correct Visual Acuity	6/36	6/60
Color Vision (Ishihara Color Plate)	30/38	24/38
Light Brightness	90%	80%
Red Saturation	90%	30%
Relative Afferent Pupillary Reflex (RAPD)	Equivocal	Grade 3
Optic Disc Examination	Pink optic disc with no features of optic disc swelling	Pink optic disc with no features of optic disc swelling
Cup-Disc-Ratio (CDR)	0.4 (vertically)	0.4 (vertically)

**Figure 2:**
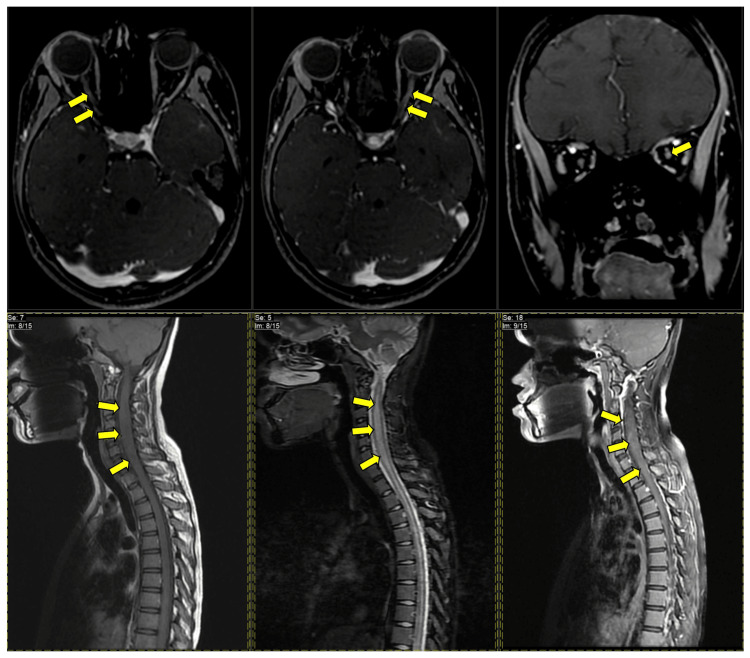
Gadolinium-contrasted T1-weighted MRI orbits (Top 3 slices-axial & coronal views) & T1-weighted, T2-flair, and gadolinium-contrasted T2 MRI spine images (in sequence) (Bottom 3 slices-sagittal view)

Results of the baseline investigations (Table 2) during admission were unremarkable. A lumbar puncture was performed and the cerebrospinal fluid (CSF) analysis results (Table 2) were unremarkable. The lumbar puncture had an opening pressure of 13mmHg. Visual evoked potential electrophysiology testing done within a week of the onset of ocular symptoms showed the absence of P100 bilaterally (Table 2). MRI showed long segment T2W/STIR spinal cord hyperintensities from the C2 to T2 level (Figure 2). Patchy contrast enhancements were also noted over bilateral retrobulbar intra-orbital optic nerves (Figure 2). Cerebrospinal fluid analysis results (Table 2) were unremarkable. 

**Table 2:** Hematological, serological, and cerebrospinal fluid (CSF) investigations and electrophysiological tests done during the initial presentation FBC: full blood count; ESR: erythrocyte sedimentation rate; CRP: C-Reactive Protein; p-ANCA: perinuclear anti-neutrophil cytoplasmic antibodies; c-ANCA: cytoplasmic anti-neutrophil cytoplasmic antibodies; HLA: human leukocyte antigen; CSF: cerebrospinal fluid; MOG: myelin oligodendrocyte glycoprotein

Laboratory Tests	Results
FBC	Normal
ESR	Normal (8mm/hour)
CRP	Normal (<5mg/L)
Tumor Markers	Negative for CA 19-9, AFP, CEA, CA125, & CA15-3
Infective Screening Tests	Non-reactive for Hepatitis B, Hepatitis C & HIV
Connective Tissue Screening	ANA, Anti-dsDNA, Complement, Rheumatoid Factor, and ENA were all within normal limits
p-ANCA & c-ANCA	Negative
Angiotensin-converting Enzyme (ACE)	Normal (24U/L)
HLA typing	HLA, DRB1 13 & 14, and HLA DQB1 05 & 06 detected
CSF appearance	Clear & colorless
CSF microscopic examination	No abnormal cells & no encapsulated yeast cells were seen
CSF leukocyte counts	Leukocyte count: 0 cells/min
CSF glucose	3.9 mmol/L
CSF protein	2.6 mg/dL
CSF cultures	No growth of bacterial or fungal colony seen
CSF oligoclonal bands IgG	Negative
Serum AQP4-IgG test (ELISA)	Negative
Serum AQP4-igG test (Cell-based assay)	Negative
Serum MOG-IgG test (Cell-based assay)	Negative
Visual evoked potential (VEP)	P100 was absent bilaterally

Her best-corrected visual acuity improved significantly to 6/12 (right eye) and 6/36 (left eye) after a five-day course of intravenous methylprednisolone (20mg/kg/day). Only at this moment, both serum aquaporin-4 antibody (AQP4-IgG) and serum myelin oligodendrocyte glycoprotein antibody (MOG-IgG) were reported to be negative(Table 2). A final diagnosis of bilateral retrobulbar optic neuritis with long-segment transverse myelitis secondary to double seronegative NMOSD was made. Plasma exchange was commenced due to incomplete visual recovery and persistent limb weakness. Catastrophically, she developed severe catheter-related septicemia after the fourth cycle of plasma exchange. Fortunately, she recovered after weeks of intravenous meropenem 1g every 8h along with ventilatory and circulatory supports. Remarkable improvements were noted in upper limb neurological functions following the completion of five cycles of plasma exchange. Upon discharge, her best-corrected visual acuity improved to 6/9 for the right eye and 6/12 for the left eye. However, she continued to experience persistent bilateral lower limb spasticity, contractures, and loss of sensation. She was discharged with azathioprine (3mg/kg/day) and oral steroids (1mg/kg/day) until the day of follow-up two weeks later. Unfortunately, she defaulted subsequent follow-up appointments despite several attempts to counsel the guardians (parents). 

## Discussion

Neuromyelitis optica spectrum disorder (NMOSD) is a rare antibody-mediated neuroinflammatory disease of the central nervous system, typically manifesting in the optic nerves, spinal cord, and other regions in the central nervous system [1-3]. The prevalence of NMOSD typically ranges from 0.5 to 4 per 100,000 individuals, with potentially higher rates among East Asians and Blacks [3]. Most individuals with NMOSD defined by the 2015 International Panel on NMO Diagnosis (2015 IPND) show seropositivity for AQP4 antibody (AQP4-NMOSD) [4]. For cases of NMOSD without AQP4 antibodies (SN-NMOSD), a more stringent set of clinical criteria, often involving additional neuroimaging findings, is necessary for diagnosis [4]. This is because detecting serum AQP4 antibodies can be challenging despite using the most sensitive laboratory techniques. Published literature reported that about 20% of samples positive for AQP4-IgG using cell-based assay could be missed by the less sensitive commercially available ELISA method [5]. Thus, the criteria also permit the diagnosis of NMOSD in individuals without AQP4 antibodies, categorized as single seronegative NMOSD (SN-NMOSD). A subset of reported seronegative NMOSD individuals are found to be positive for myelin oligodendrocyte glycoprotein (MOG) antibodies [4]. This case exemplifies individuals with double seronegative NMOSD (DN-NMOSD) phenotype for which they remain persistently negative for both AQP4 and MOG antibodies.

DN-NMOSD poses significant diagnostic and therapeutic challenges in clinical practice. Its classification within the neuro-inflammatory diseases of the central nervous system remains unknown. The proportion of DN-NMOSD among NMOSD patients varies from 0 to 79% [6]. While the pathophysiology of DN-NMOSD might differ between patients, there may be some common features as evidenced by shared clinical and radiological characteristics [6]. It remains unclear whether DN-NMOSD is primarily an astrocytopathy similar to AQP4-NMOSD [6]. 

Diagnosing NMOSD (AQP4-NMOSD, SN-NMOSD, and DN-NMOSD) involves recognizing the clinical features, MRI findings, and serum antibody markers. However, there are other proven investigative modalities that can be helpful. Mixed pleocytosis (lymphocytes, neutrophils, and monocytes) in the cerebrospinal fluid (CSF) is typically found in NMOSD patients during an acute attack [1]. Oligoclonal IgG bands are present in only 10-30% of NMOSD patients, contrasting with 90% of MS patients [7]. Elevated glial fibrillary acidic protein (GFAP) in the CSF is a useful biomarker of astrocytic destruction during NMOSD acute attacks [6]. Visual evoked potential (VEP) electrophysiology amplitudes reflect the number of functioning optic nerve fibers, and its latency prolongations reflect optic nerve demyelination. VEP may demonstrate the absence of response along with decreased amplitude in NMOSD (lasting at least three months after the onset) [8]. Optical coherence tomography (OCT) in NMOSD reveals significantly greater reductions in retinal nerve fiber layer thickness (mean 63.6 vs 88.3 μm, p < 0.0001) and macular volume (5.83 vs 6.38 mm3, p = 0.001) as compared to MS [9]. Severe thinning of the retinal nerve fiber layer (RNFL) in NMOSD likely results from greater degeneration of the retinal ganglion cell axons. Unfortunately, OCT analysis was not performed for this case.

Evidence-based guidance for the management of DN-NMOSD is limited. Many experts manage acute attacks in a similar manner to antibody-positive patients, employing conventional therapies used for AQP4-NMOSD and MOGAD disease to suppress the immune system in recurring cases. For acute attacks, high-dose corticosteroids are the first-line therapy, often starting with 1000mg of methylprednisolone intravenously for five days [4]. Plasma exchange (PLEX) has been suggested as a rescue treatment modality for patients with severe symptoms not responding to steroids [6]. However, hemodynamic instability, anaphylaxis, and catheter-related septicemia are the life-threatening adverse effects of PLEX.

Mycophenolate mofetil (MMF: 2-3g/day) and azathioprine (AZA: 2.5-3mg/kg) are common long-term immunosuppressants used in all NMOSD cases to prevent relapses [4]. Oral prednisolone (5-10mg) is frequently prescribed long-term as the combination may be more protective than MMF/AZA alone and it also covers the period for AZA/MMF to take full effect [4]. The B-cell depleting monoclonal antibody, rituximab (RTX), served as a second-line medication, reducing relapse rates up to 88.2% [6].^ ^Significantly, several immunomodulators used in MS are ineffective and may exacerbate relapses in AQP4-NMOSD patients (i.e. interferon-β, glatiramer acetate, natalizumab, alemtuzumab, and fingolimod) [4,6]. To date, there are no approved agents specifically for preventing relapses in DN-NMOSD [6].

If left untreated, approximately 50% of NMOSD patients may become wheelchair-bound and blind, and up to a third may succumb within five years of their first attack [4]. Follow-up analysis suggests poor recovery from optic neuritis in NMOSD compared to other idiopathic demyelinating optic neuritis [8]. Attacks in patients with DN-NMOSD cause similar or worse residual disability as compared to AQP4-NMOSD [6].Twenty-five percent of DN-NMOSD patients have been reported to be left with severe visual impairment [7]. It is depressing to encounter young patients with such disfiguring contractures and residual disability. The final outcomes may be different if there had been better awareness of the presentation of this rare disease among the public as well as the healthcare workers. 

## Conclusions

In conclusion, we report a rare case of DN-NMOSD, emphasizing its diagnostic and therapeutic significance. Its diagnostic criteria are more stringent as compared to SN-NMOSD and AQP4-NMOSD. Healthcare workers should be vigilant and well-aware of the typical presentations of this rare disease. Besides utilizing the diagnostic criteria mentioned in the International Panel for NMO Diagnosis of 2015, it is prudent to analyze the findings in CSF, VEP, and OCT. Prompt management of DN-NMOSD is crucial as it can result in similar disabilities as in AQP4-NMOSD.
